# Cardiovascular Risk Associated With Gender Affirming Hormone Therapy in Transgender Population

**DOI:** 10.3389/fendo.2021.718200

**Published:** 2021-09-30

**Authors:** Gloria Aranda, Irene Halperin, Esther Gomez-Gil, Felicia A. Hanzu, Núria Seguí, Antonio Guillamon, Mireia Mora

**Affiliations:** ^1^ Group of Endocrine Disorders, Institut d’Investigacions Biomèdiques August Pi I Sunyer- Hospital Clinic, Barcelona, Spain; ^2^ Endocrinology Department, Hospital Clinic, Barcelona, Spain; ^3^ Psychiatry Department, Hospital Clínic, Barcelona, Spain; ^4^ Departamento de Psicobiologia, Universidad Nacional de Educación a Distancia (UNED), Madrid, Spain

**Keywords:** transgender women, transgender men, transgender population, gender affirming hormone therapy, cardiovascular risk

## Abstract

Transgender men and women represent about 0.6 -1.1%% of the general population. Gender affirming hormone therapy (GAHT) helps ameliorate gender dysphoria and promote well-being. However, these treatments’ cardiovascular (CV) effects are difficult to evaluate due to the limited number of extensive longitudinal studies focused on CV outcomes in this population. Furthermore, these studies are mainly observational and difficult to interpret due to a variety of hormone regimens and observation periods, together with possible bias by confounding factors (comorbidities, estrogen types, smoking, alcohol abuse, HIV infection). In addition, the introduction of GAHT at increasingly earlier ages, even before the full development of the secondary sexual characteristics, could lead to long-term changes in CV risk compared to current data.

This review examines the impact of GAHT in the transgender population on CV outcomes and surrogate markers of CV health. Furthermore, we review available data on changes in DNA methylation or RNA transcription induced by GAHT that may translate into changes in metabolic parameters that could increase CV risk.

## Introduction

Transgender people represent about 0.6 – 1.1% of the general population, 0.7 and 1.1% of people assigned male at birth, and 0.6 and 0.8% of people assigned female at birth, as described in an European study of T’Sjoen et al. (1). Gender affirming hormone therapy (GAHT) helps ameliorate gender dysphoria by changing the physical appearance in accordance with gender identity and expression and promote well-being (2). However, this therapy’s cardiovascular (CV) effects are difficult to evaluate because most of the studies are observational and can have a possible bias due to confounding factors such as comorbidities, variety of hormone regimens, smoking, alcohol abuse or HIV infection. Cardiovascular disease (CVD) is the main cause of death for transgender people undergoing GAHT, although suicide is still the leader of all-cause mortality (3). However, for transgender women, the risk of death from CVD is 3-fold higher than for ciswomen and cismen and has been associated with the use of ethinyl estradiol (EE) (2, 4). Moreover, the introduction of GAHT at increasing earlier ages may lead to changes in CV risk compared to current data.

This review examines the impact of GAHT in transgender people on CV outcomes and surrogate markers of CV health. Furthermore, we review available data on changes in DNA methylation or RNA transcription induced by GAHT that may translate into changes in metabolic parameters that could increase CV risk.

## Methods

We performed a review to evaluate CV health in transgender population. We searched in PubMed/MEDLINE databases for articles with this topic, we included articles published until April 2021, and we limited the search to English language articles. The Keywords were transgender, transgender men, transgender women, hormone therapy, GAHT, estrogen, antiandrogen, progesterone, testosterone, cardiovascular disease, and cardiovascular risk factors.

We included retrospective, observational, cohort, cross-sectional studies, population survey of transgender individuals, with a minimum population size of 100 individuals and a follow-up of 1 year, with GAHT regardless of doses or gender affirming surgery in which CV outcomes (thromboembolism, myocardial infarction, stroke) and surrogate markers of CV risk have been assessed.

## Feminizing Hormone Therapy in Transgender Women

Current evidence from Europe and America suggests that GAHT initiated and monitored under medical supervision is associated with very low rates of adverse events (5, 6). However, factors associated with a higher risk of thromboembolic conditions, such as smoking, obesity, and sedentary lifestyle, should be evaluated in transgender women prior to initiating GAHT and modified if possible. In certain cases, using the transdermal route and anticoagulant treatment should be considered to prevent thromboembolisms. In addition, other diseases such as coronary artery and cerebrovascular disease, hormone-sensitive cancers, hyperprolactinemia, hypertriglyceridemia, and cholelithiasis should be assessed, as these conditions can be exacerbated by estrogen and may be considered relative contraindications for GAHT (7, 8). Moreover, information related to fertility preservation should be provided, and options for preservation should be discussed and offered before starting the medication.

### Types of Hormonal Therapy in Transgender Women

There are two main classes of medications used in transgender women: estrogenic therapies and androgen-lowering hormone therapies. **Table 1** shows the most frequent regimens used nowadays.

**Table 1:** Gender affirming hormone therapy.

Hormone	Route	Doses	Considerations
**Transgender women**			
Estradiol valerate Estradiol Estradiol valerate or cypionate	Oral Transdermal patch. New patch placed every 3 – 5 d Parenteral	2 – 6mg/d 0.025 – 0.2mg/d 5 – 30mg IM every 2wk 2 – 10mg IM every wk	<45 years >45 years
**Anti-androgens** Spironolactone Cyproterone acetate	Oral Oral	100 – 300mg/d 25 mg/d	Preferred in USA Preferred in Europe
Triptorelin (GnRH agonist)	SC	3.75mg/monthly 11.25mg/3 monthly	Preferred in UK instead of antiandrogens
**Transgender males**			
Testosterone Testosterone enanthate or cypionate Testosterone undecanoate Testosterone gel 1.6% Testosterone patch	Parenteral Parenteral Transdermal Transdermal	100 – 200mg every 2-4 wk or 50% per 1-2 wk 1000mg every 12 wk 50 – 100mg/d 2.5 – 7.5mg/d	

SC, subcutaneous; WK, week.

In relation to estrogenic therapies, EE is a synthetic estrogen widely used in Europe prior to 2003 (9). However, given recent safety concerns about its prothrombotic potential and its possible role in CV disease, most clinicians have now switched to oral, cutaneous, or IM estradiol valerate (7). Studies that compare the long-term safety and effectiveness among the different formulations of estradiol are lacking. The Endocrine Society guidelines recommend titrating the doses to serum estradiol levels at 100-200pg/ml (367.1-734.3 pmol/l) (7).

In general, androgen-lowering therapies are required to reduce testosterone levels into the female range. One of the most prescribed androgen-lowering medications is oral cyproterone acetate (CPA) (5, 10). CPA is an androgen receptor blocker but also has some progesterone-like activity (11). Due to reports of increased incidence of meningiomas (12–14), association with depression, and increased risk of hyperprolactinemia with CPA use, the maximum recommended dose is 25mg daily (15, 16). The study by Kuijpers et al. has observed that the 10mg dose of CPA is as effective as higher doses but with fewer side effects (17). In UK, Italy, and Netherlands, transgender women are now treated with GnRH agonist (GnRHa) to lower testosterone levels (18). GnRHa substitutes the pulsatile physiological release of GnRH by a continued release of the GnRHa administrated and inhibits the secretion of FSH/LH from the pituitary to the testicle/ovary. Gosereline and leuprolide have been used instead of CPA to reduce testosterone levels and lower adverse reactions (7). Spironolactone lowers testosterone synthesis and action at the androgen receptor and is also an antagonist of the mineralocorticoid receptor and potassium-sparing diuretic. Flutamide also has antiandrogenic effects, but it is not recommended (7, 18, 19).

Progesterone therapies such as medroxyprogesterone have been used to reduce testosterone concentrations in transgender women (19). Some may ask for progesterone to enhance breast development; however, clinical evidence does not support this effect (20). Furthermore, there are concerns regarding the potential increased risk of thromboembolism and stroke found in cisgender women taking progesterone (21, 22).

Finasteride therapy (5α-reductase inhibitor) has effectively improved hair loss in transgender women with androgenic alopecia without significant side effects. Nevertheless, the routine use of 5α-reductase inhibitors has been limited over previous concerns of long-term sexual dysfunction and depression reported in cisgender men (23, 24).

### Physical Changes in Transgender Women

The aim of GAHT in transgender women is to induce feminine and reduce masculine physical traits. The development of breast tissue is one of the most expected changes in transgender women and is associated with improvement in body discomfort score (25). However, less than 20% of transgender women reach Tanner breast stage 4 to 5 after 24 months of hormone therapy; therefore, mammoplasty is often requested. Several studies show that a plateau is achieved within the first 6-9 months of treatment (26–28). Recent data suggests that sustained breast development was observed during a period of three years of follow-up with a more lateral and caudal position compared to ciswomen (28). Testicular volume decreases ~ 60% after 24 months of GAHT is also observed, as well as reduction of erections and ejaculation (25). An increase in body weight was associated with an increase in body fat, specifically in the gynoid regions, and a reduction in lean body mass (25, 29–31). Facial hair diminished and the Ferriman-Gallwey scores improved after two years of GAHT, as well as the hair loss pattern (25). Voice changes are seldom observed; therefore, transgender women will have to look for voice therapy or phonosurgery (32, 33).

### Metabolic Changes in Transgender Women

The metabolic effects of estrogen therapy are focused on liver function alterations and lipid parameters. Hepatic lipase activity decreases by 64% and lipoprotein lipase by 23%. Hepatic lipase decreases HDL-cholesterol (HDL-c) levels and increases the formation of small, dense LDL (LDL-c) highly atherogenic. Reduction of hepatic lipase levels with estrogen may reduce the formation of LDL-c (34). However, the use of estrogen therapy in transgender women showed no statistically significant difference in total cholesterol, serum LDL-c or HDL-c, but an increase in plasma triglyceride levels after 24 months was found (35). Conflicting data seem to show unmodified or reduced insulin sensitivity, with unaltered fasting glucose and stable or increased blood pressure (34). Estrogen therapy in transgender women has been associated with reduced plasma homocysteine levels, independently of the route of administration (36, 37). The impact of estrogens on prothrombotic status remains unclear. While some studies with EE in combination with CPA, but not with transdermal estradiol, show an increase in C-reactive protein (CRP) and decreased tissue plasminogen activator; other studies have shown no effect on CRP (38, 39).

### Cardiovascular Outcomes in Transgender Women

Data in transgender women receiving estrogen therapy are limited to observational and cohort studies. However, retrospective studies have shown a higher incidence of thromboembolic events in transgender women with EE and CPA compared to a similar reference group of the population (40) and using equine estrogens compared to estrogen valerate or EE when mammoplasty was performed (16). **Table 2** shows the studies that evaluated CV outcomes and mortality associated with GAHT.

**Table 2:** Cardiovascular outcomes.

Author	Country/Year	n	Study	GAHT	Follow-up	Time of GAHT	CV Outcomes (n)	Mortality
**Asscheman H et al.** (40)	Netherlands 1989	303 TW 122 TM	Retrospective study	EE 100ug+ CPA 100mg Long-acting testosterone ester 250mg/2wk Oral testosterone undecanoate 120 -160mg/d	14 years	TW 4.4yr TM 3.6yr	VT/PE (29) TW Weight ↑ >10% MI (2) TW	No CV mortality
**Van Kesteren PJ et al.** (41)	Netherlands 1997	816 TW 293 TM	Retrospective study	Oral estrogens + anti-androgens. Transdermal estradiol			VT/PE TW No morbidity in TM	Total mortality was no higher than in the general population
**Asscheman H et al.** (4)	Netherlands 2011	966 TW 365 TM	Cohort study	EE or conjugated estrogens (until 1989). Transdermal E2 Estradiol valerate 2-4mg/d CPA 100mg/d Spironolactone 100 – 200mg/d. Testosterone ester IM 250mg/2wk Oral testosterone undecanoate 160-240mg/d Transdermal testosterone 50mg/d Lynestrenol (uterine bleeding persisted)	18.5 years	TW 19.4yr TM 18.8yr	Ischemic heart disease 18 TW 1 TM Cerebrovascular accidents 5 TW 0 TM	SMR TW 1.51 (1.47-1.55) SMR TM 1.12 (0.89-1.59)
**Dhejne C et al.** (3)	Sweden 2011	191 TW 133 TM	Cohort study	Not available	30 years		Not available	HR 2.5 (1.2-5.3)
**Wierckx K et al.** (42)	Belgium 2012	50 TW 50 TM	Cross-sectional study	Estrogen + CPA Testosterone	10 years	TW 6.3yr TM 8.7yr TM	Only TW TV/PE (4) TIA (1) MI (1)	Not available
**Wierckx K et al.** (43)	Belgium 2013	214 TW 138 TM	Case-control study	Transdermal estradiol gel/patch Estradiol valerate 2mg EE 50mcg Oral contraceptive Testosterone ester IM 2-3wk Transdermal testosterone 50mg/d Oral testosterone undecanoate	22 years	TW 7.7yr TM 9.7yr	TW VT/PE MI TIA/CVD T2DM TM VT/PE T2DM	9 TW 1 TM
**Wierckx K et al.** (5)	Belgium 2014	53 TW 53 TM	Prospective multicenter study	<45yr 50mg CPA + 4mg valerate estradiol >45yr 50mg CPA + 100ug/24hs transdermal 17B estradiol IM Testosterone undecanoate every 3 months	12 months	12 months	No severe adverse events	No deaths were observed
**Nokoff N et al.** (44)	USA 2018	369 TW 239 TM 156 GNC	Secondary Data analysis based on 2015 Behaviors Risk Factor Surveillance System Survey (CDC) Cross-sectional study	Not available	–	–	TW HTA 29.2% MI 5.5% Angina or CHD 3.5% Stroke 2.6% TM HTA 25.2% MI 2% Angina or CHD 3.1% Stroke 2.3% No differences in GNC between cisgender men and women	
**Alzahrani T et al.** (45)	USA 2019	1842439 1267 TM 1788 TW	Combined data of Behavioral Risk Factor Surveillance System (BRFSS) (CDC) Cross-sectional study	Not available	-	-	MI TW OR 2.56 vs CIS W No increase vs CIS M TM OR 2.53 vs CIS M OR 4.90 vs CIS W	Not available
**Nota NM et al.** (46)	Netherlands 2019	2517 TW 1358 TM	Retrospective study	Not available	43 years	TW: 22.83yr TM: 11.03yr	TW Stroke (29) MI (30) VT (73) TM Stroke (6) MI (11) VT (2)	Not available
**Scheres JLL et al.** (47)	European Network for the Investigation of Gender Incongruence (ENIGI) 2021	92 TW 100 TM	Longitudinal study Baseline and 12 months after GAHT	Oral estrogen Transdermal estrogen Anti-androgen therapy + estrogen oral Anti-androgen monotherapy Transdermal testosterone IM testosterone	12 months	12 months	TW ↑FIX ↑FXI ↓pC ↓ activated protein C resistance	Not available

CHD, Coronary heart disease; CIS M, cisgender men; CIS W, cisgender women; CPA, Cyproterone acetate; CV, cardiovascular; EE, Ethinyl Estradiol; FIX, Factor IX; FXI, Factor XI; GAHT, gender affirming hormone therapy; HR, Hazard ratio; MI, Myocardial Infarction; pC, C protein; TIA, Transient ischemic attack; TM, Transgender men;TW, Transgender women; VT/PE, venous thrombosis, embolism pulmonary; ↑: increased; ↓: decreased.

A systematic review and meta-analysis of CV outcomes in transgender people reported few cases of myocardial infarction (MI), stroke, or venous thrombosis; however, the incidence was higher in transgender women compared to transgender men (35, 40).

In 2018, a nationwide US survey was distributed across 22 states and included questions about the transgender condition; 0.6% of those surveyed identified as transgender people. The study found that transgender women reported higher MI than cisgender women (OR 2.9; 95% CI, 1.6 to 5.3; p<0.001) but with no differences when compared to cisgender men (44).

In addition, a survey conducted by the Centers for Disease Control and Prevention, with 1.8 million participants, also observed that all transgender individuals receiving GAHT had significantly higher rates of MI compared to their cisgender counterparts; after adjusting for CVD risk factors, transgender women had more than a two-fold increased risk in MI compared to cisgender women. Transgender women had no significant difference in MI risk compared to cisgender men (45).

A Dutch study of 2517 transgender women using estrogen followed for an average of 9 years found twice as many strokes and MIs as in cisgender women and almost twice as many strokes and no difference in MIs compared to cisgender men; also a five-fold and four-fold increase risk in thromboembolic events compared to both ciswomen and cismen, respectively (46).

## Masculinizing Hormone Therapy in Transgender Men

Masculinizing GAHT in transgender men favors male secondary sex characteristics and minimizes feminine traits. Transgender men must be informed of the necessity of lifelong therapy with testosterone, its possibilities, risks, consequences, and limitations. Information related to options for the preservation of fertility should be provided before starting GAHT (7). Pregnancy contraindicates testosterone therapy, and relative contraindications include severe hypertension, sleep apnea, and polycythemia. Erythrocytosis, sleep apnea, and congestive heart failure can be exacerbated by testosterone therapy (7).

### Types of Hormonal Therapy in Transgender Men

The main GAHT used to induce virilization is testosterone. Different testosterone formulations may be available depending on geographical location (**Table 1**). Most prescribed are injectable testosterone esters. Both parenteral and transdermal administration of testosterone are equally effective to achieve masculinization and serum testosterone values in the range of 300 - 1000ng/dl (10.4–37.4 nmol/l) in transgender men. Serum testosterone levels in injectable formulations are measured between administrations, although clinicians may choose to measure serum testosterone 24hs after injection and prior to the next dose (7).

More recently, the subcutaneous administration of testosterone was shown to be effective and preferred by transgender men at a median dosage of 75mg weekly (48). In Pelusi et al. study, the effects of three different testosterone formulations (gel, cypionate, and undecanoate) were evaluated at baseline. After 12 months of treatment, no differences were found regarding short-term safety, compliance, body composition, or metabolic parameters (49).

If menstrual bleeding does not stop after initiation of testosterone, a progestational agent, such as oral lynestrenol at 5 to 10mg daily or medroxyprogesterone at 5 to 10mg, might be considered (7, 50).

### Physical Changes in Transgender Men

Menses discontinuation, clitoris enlargement, and lower-pitched voice are some of the changes aimed by transgender men (7, 51, 52). In addition, therapy will enhance a more masculine musculature, body shape with an increase in body weight, a decrease in body fat, and an increase in lean mass as well as grip strength (51–55). Testosterone therapy has been associated with increases in the Ferryman-Gallwey hirsutism scores. However, after 12 months, facial and abdominal hair do not reach diameters found in cisgender males. An increase in acne and alopecia is often observed as some of the side effects (51, 52, 54).

### Metabolic Changes in Transgender Men

Lipid parameters are adversely modified by testosterone therapy in transgender men. A recent meta-analysis of the available data demonstrated no change in total cholesterol or LDL-c. Still, there was a minor increase in triglyceride and a decrease in plasma HDL-c levels, both of which are pro-atherogenic (55, 56). Another meta-analysis showed a progressive change in lipid parameters over 24 months with higher triglycerides levels compared with baseline; statistically significant serum LDL-c increase and HDL-c decrease were also observed, with no statistically significant differences in total serum cholesterol level (56). Testosterone therapy has no effect on fasting glucose, fasting insulin, or glucose utilization. However, transgender men were found to have decreased adiponectin, which is associated with insulin resistance and higher CV risk (34). Regarding blood pressure, the results of various studies are contradictory (57). Testosterone therapy increases plasma homocysteine levels in transgender men, which could have a negative impact on CV risk. After one year of hormonal treatment, transgender men presented increased homocysteine and leucocytes levels, with an increase in mean maximum carotid intimal media thickness (36, 54).

### Cardiovascular Outcomes in Transgender Men

Present evidence regarding testosterone therapy and CV disease risk in transgender men is controversial. Several studies have observed that despite the negative effects of testosterone therapy on surrogate risks factors of CV disease, these do not translate into a significant effect on CV outcomes (**Table 2**). Furthermore, no elevated rates of CV deaths have been observed when compared with cisgender men and women at short and medium follow-up (30, 35, 40, 56).

In a cross-sectional study of 50 transgender men on testosterone therapy during an average of 10 years, no subject had experienced MI, stroke, or deep venous thrombosis (57). In a similar case-control study, 138 transgender men on testosterone therapy for an average of 7.4 years showed low CV morbidity. In transgender men, MI was higher when compared to cisgender women, but there was no difference when compared to cisgender men. After adjustment for CV risk factors, however, the study demonstrated that transgender men had an increased risk for MI compared to both cisgender populations. This study emphasizes the importance of additional CV risk factors such as smoking, reduced exercise, diabetes, and non-Caucasian ethnic origin, all of which were seen in higher numbers in the transgender population (35, 43). Similar data in the Dutch analysis of 1358 transgender men using testosterone followed for an average of 8 years, which found three times more MIs as in cisgender women with no differences compared to cisgender men and no differences in stroke compared to cisgender women or men (46).

There have been reports of a possible link between testosterone replacement therapy use and increased venous thromboembolism risk; however, these studies were criticized for including data on avascular necrosis of the femoral head, which are not classically viewed as venous thromboembolic events. Extensive epidemiological studies have demonstrated that there is no link between testosterone therapy and thromboembolism risk (46, 58).

## Hormonal Treatment in Adolescents

The treatment in adolescents is generally based on two phases: the first phase consists of suppressing the gonadal axis once puberty has commenced (Tanner 2-3); The second phase is the introduction of the GAHT. Gonadal suppression is generally performed with GnRHa such as gosereline, leuprolide, triptoreline, and histrelin; it provides the adolescent and its family with the time and the space to explore the gender identity before the treatments starts which can imply irreversible changes. Suppression also improves the anxiety for developing the secondary sexual characters associated with the gender assigned at birth (7, 59). Scarce information is available concerning the metabolic effects of suppressive treatment; however, a decrease in height velocity in both transgender girls and boys is observed, as well as an increase in body mass index in comparison to the gender assigned at birth, with an increase in fat body mass and a decrease in lean body mass during the first year, that is stabilized afterwards. No effects on lipid or carbohydrate metabolism have been described (59). However, long-term CV effects are still unknown, so a healthy lifestyle and no smoking are encouraged. The effects of GnRHa on bone structure are still in debate. Data suggest that bone mineral density is preserved, but z-score decreases (more in transgender girls than boys) with improvement after GAHT introduction (60). Some side effects must be monitored, such as flushing, headache, mood changes, and hypertension (triptoreline) or intracranial hypertension (rare and associated to leuprolide). Other less effective alternatives used as a suppressive treatment are CPA in transgender girls or medroxyprogesterone to suppress menses in transgender boys (59–63).

GAHT introduction is generally recommended around the age of 16, although it can be considered around the ages of 14-16, even though there are very few published studies of being administered between the ages of 13.5 and 14 (7). There are two treatment regimens. In the case that GnRHa was introduced early in pubertal development, the puberty of the desired gender is induced by slow increasing doses of testosterone or estradiol, that are modified every six months. In the case that GnRHa began late in puberty, the suppression lasts about 3-6 months and GAHT begins at higher doses, with a faster increase to achieve maintenance dose (7, 62).

Since the long-term effects of both suppressive and GAHT treatment are uncertain, adolescents must be encouraged to adopt a healthy lifestyle, increase exercise, avoid tobacco, and keep regular check-ups with the endocrinologist for the monitorization of liver and renal function, lipids, and glucose.

## Molecular Effects of Gender Affirming Hormone Therapy

Basic research has found Androgen Receptors (AR) and Estrogen Receptors (ERs) in endothelial cells, suggesting that masculinizing and feminizing hormones have a direct impact on the vascular endothelium. Testosterone and estrogen bind to these receptors producing an increase in transcription of athero-protective genes and a downregulation of pro-atherogenic genes, which could be associated with a decrease in CV risk (64). In the work carried out by our group, we observed an increase in the ER methylation pattern in transgender men after 12 months of GAHT, an increase in AR methylation pattern in transgender women after 12 months of estrogenic treatment. Regarding the expression analysis, AR expression was significantly decreased in transgender men. AR, ER methylation were correlated with anthropometric, metabolic, and hormonal parameters, supporting that GAHT is associated with epigenetic changes that might affect the response to treatment with sex steroids (65). More recent data has also shown that GAHT modified the methylation pattern of ER, more similar to their gender (66). Therefore, research in methylation and expression of AR and ER may help to understand the different effects of GAHT in physical, metabolic, and CV outcomes in transgender people.

## Discussion

Several studies have been published concerning the metabolic effects of GAHT in both transgender women and men. However, data for metabolic effects are often contradictory and inconclusive. The main reasons are the observational and retrospective nature of studies, including populations with diverse hormonal regimens without medical supervision; most of them include EE at high doses in transgender women; the influence of possible bias by confounding factors (comorbidities, smoking, alcohol abuse, or HIV infection) (2–5, 9, 35, 40, 46).

In transgender women, recent regimens have excluded EE and suse oral and transdermal estradiol associated to CPA or other androgen-lowering agents under endocrinological control. Unfortunately, prospective data are still limited, and it is uncertain if these changes may improve CV and thromboembolic risk (2–7, 35, 40, 45). However, recent data suggest a higher risk of MI compared with ciswomen with no differences in comparison to cismen (44–46).

In transgender men, previous data suggest a lower risk of CV events in comparison to birth–assigned males, probably due to testosterone introduction at later ages and the possible protective effect of endogen estrogens before GAHT (2–7, 35, 40, 44–46). A prospective study performed by our group showed an impairment of lipid profile and an increase of homocysteine and leucocytes count, as well as a higher mean-maximum common intima-media thickness after 12 months of GATH (55). Recent data suggest a higher risk of MI in transgender men in comparison with ciswomen.

The introduction of GAHT at increasingly earlier ages, even before the full development of the secondary sexual traits, could lead to long-term changes in CV risk compared to current data and bring it closer or higher to the known risk of the identified gender (59–63). Future research is essential to find out this risk.

Moreover, non-binary transgender population may yearn for a more personalized treatment with a partial suppression of the traits associated with the gender assigned at birth and the development of some of the traits of the other gender. No clear regimens have been established, and no information is available concerning the effects on CV risk. Hence, future research in this group is necessary to ascertain their risk of CV illness.

In conclusion, future research should join forces to obtain data from prospective controlled studies, including larger samples. Therefore, studies should consider the introduction of GATH at progressively younger ages as well as the voice of non-binary transgender population in order to improve the knowledge of the CV effects of hormone therapy in these situations.

## Author Contributions

Conceived and designed the review: MM. Writing the manuscript: GA, MM, IH and NS. Revised the manuscript and contributed significantly to the intellectual value of the paper: IH, FH, EG-G, and AG. All authors contributed to the article and approved the submitted version.

## Funding

This review has been possible with the economical help of the project PGC2018-094919-B-C21.

## Conflict of Interest

The authors declare that the research was conducted in the absence of any commercial or financial relationships that could be construed as a potential conflict of interest.

## Publisher’s Note

All claims expressed in this article are solely those of the authors and do not necessarily represent those of their affiliated organizations, or those of the publisher, the editors and the reviewers. Any product that may be evaluated in this article, or claim that may be made by its manufacturer, is not guaranteed or endorsed by the publisher.
